# Brain Metabolite Levels in Sedentary Women and Non-contact Athletes Differ From Contact Athletes

**DOI:** 10.3389/fnhum.2020.593498

**Published:** 2020-11-26

**Authors:** Amy L. Schranz, Gregory A. Dekaban, Lisa Fischer, Kevin Blackney, Christy Barreira, Timothy J. Doherty, Douglas D. Fraser, Arthur Brown, Jeff Holmes, Ravi S. Menon, Robert Bartha

**Affiliations:** ^1^Department of Medical Biophysics, Robarts Research Institute, Centre for Functional and Metabolic Mapping, Western University, London, ON, Canada; ^2^Molecular Medicine Research Laboratories, Robarts Research Institute, Western University, London, ON, Canada; ^3^Department of Microbiology and Immunology, Western University, London, ON, Canada; ^4^Fowler Kennedy Sport Medicine Clinic, Department of Family Medicine, Western University, London, ON, Canada; ^5^Physical Medicine and Rehabilitation, Western University, London, ON, Canada; ^6^Paediatrics Critical Care Medicine, London Health Sciences Centre, London, ON, Canada; ^7^Department of Anatomy and Cell Biology, Western University, London, ON, Canada; ^8^School of Occupational Therapy, Western University, London, ON, Canada

**Keywords:** magnetic resonance spectroscopy, glutamine, concussion and sports, exercise, sub-concussive head impact, sub-concussion

## Abstract

White matter tracts are known to be susceptible to injury following concussion. The objective of this study was to determine whether contact play in sport could alter white matter metabolite levels in female varsity athletes independent of changes induced by long-term exercise. Metabolite levels were measured by single voxel proton magnetic resonance spectroscopy (MRS) in the prefrontal white matter at the beginning (In-Season) and end (Off-Season) of season in contact (*N* = 54, rugby players) and non-contact (*N* = 23, swimmers and rowers) varsity athletes. Sedentary women (*N* = 23) were scanned once, at a time equivalent to the Off-Season time point. Metabolite levels in non-contact athletes did not change over a season of play, or differ from age matched sedentary women except that non-contact athletes had a slightly lower *myo*-inositol level. The contact athletes had lower levels of *myo*-inositol and glutamate, and higher levels of glutamine compared to both sedentary women and non-contact athletes. Lower levels of *myo*-inositol in non-contact athletes compared to sedentary women indicates long-term exercise may alter glial cell profiles in these athletes. The metabolite differences observed between contact and non-contact athletes suggest that non-contact athletes should not be used as controls in studies of concussion in high-impact sports because repetitive impacts from physical contact can alter white matter metabolite level profiles. It is imperative to use athletes engaged in the same contact sport as controls to ensure a matched metabolite profile at baseline.

## Introduction

There is growing concern that repetitive mild traumatic brain injuries from concussion put athletes at an increased risk of cognitive dysfunction (Montenigro et al., 2017; Gangolli et al., 2019) and long-term neurodegeneration (McCrory et al., 2013). Much less is known about the effect of repeated sub-concussive head impacts from physical contact in sport, particularly in female athletes (McGroarty et al., 2020). Such impacts are generally thought to be inconsequential, but may lead to subtle changes in brain metabolism and function (Manning et al., 2020). Detecting such effects must account for potential beneficial changes induced by exercise. Importantly, the proper interpretation of brain changes following concussion also requires knowledge of whether repetitive impacts from physical contact can modulate brain metabolism and function.

Although many different non-invasive imaging modalities have been used to study changes in the brain following concussion, magnetic resonance spectroscopy (MRS) provides neurochemical information that can be directly related to the known neurometabolic cascade (Giza and Hovda, 2014). By tracking MRS metabolite measurements over a sports season both the effects of exercise and sub-concussive impacts can also be studied. Several previous studies have measured metabolite changes in athletes during their athletic season, to investigate the effect of cumulative sub-concussive impacts (Poole et al., 2014, 2015; Tremblay et al., 2014; Mayer et al., 2015; Churchill et al., 2017; Bari et al., 2018; Lefebvre et al., 2018; Panchal et al., 2018). In contact athletes, elevated *myo*-inositol (Lefebvre et al., 2018) and reduced Glx (glutamate + glutamine) (Poole et al., 2014, 2015; Lefebvre et al., 2018) have been reported in gray matter (GM) in both sexes (Age 15–27), as well as reduced choline, and Cr (creatine) (Poole et al., 2014, 2015) in GM in males (Age 15–18). Moreover, elevated Glx/Cr (Bari et al., 2018) in the GM of females (Age 14–18) and reduced *N*-acetyl aspartate (Mayer et al., 2015; Panchal et al., 2018) in white matter (WM) and GM in both sexes (Age 15–33) have also been observed after a season of play. Taken together, these studies demonstrate alterations in the metabolite profile of contact athletes in the absence of concussion. However, the majority of these studies examined male participants or did not differentiate between males and females. Additionally, there is a lack of consensus with regards to the definition of a non-contact control group in the literature (e.g., classifying basketball, tennis, or softball as non-contact sports). A previous study by our group in the prefrontal white matter of female varsity rugby players (Schranz et al., 2018), found reduced glutamine in non-concussed players at the end of their season in addition to reduced glutamine in concussed athletes. However, this study did not include a group to control for an exercise effect across seasons of play.

With regards to exercise, previous studies have demonstrated that the brains of athletes differ from non-athletic, sedentary controls, likely due to neuroanatomical adaptations and plasticity in response to long-term training (Wang et al., 2013; Huang et al., 2015). For example, a global increase in brain non-oxidative metabolism of carbohydrate substrates has been reported to induce changes in proton MRS measured metabolites (Maddock et al., 2016). Specifically, studies have found increased lactate and Glx, as well as acute modulation of glutamate and GABA (γ-aminobutyric acid) after vigorous exercise (Maddock et al., 2011, 2016) in the GM of both males and females (Age 18–38). Whether, these changes translate into a chronic adaptation in metabolite levels, such as over the course of a sports season, is not known. Perhaps closest to this paradigm is a study by Gonzales and colleagues (Gonzales et al., 2013) who examined the effect of endurance training in both sexes (Age 46–58) and found elevated *N*-acetyl aspartate/Cr and choline/Cr in GM in the endurance trained group compared to normal healthy controls. However, this study included only athletes >40 years of age and did not report the duration of endurance training. Therefore, the effects of long-term exercise on MRS measured metabolites remains to be determined.

The main objective of the current longitudinal study was to determine whether white matter metabolite levels in varsity rugby athletes engaged in repetitive physical contact differed from varsity rowers, varsity swimmers, and sedentary individuals who were not involved in repetitive physical contact. Specifically, we were interested in examining *N*-acetyl aspartate, choline, creatine and *myo*-inositol as these metabolites have been previously shown to change following contact play, as well as glutamine due to the previous finding of glutamine changes by our group in female rugby players. Use of these groups permitted the investigation of the effects of physical contact, while accounting for the effects of exercise. It was hypothesized that there would be no differences in metabolite profile between non-contact female athletes and sedentary women, but an altered metabolite profile would exist in the female contact athletes due to repetitive head impacts.

## Materials and Methods

### Participants

Sedentary females and female athletes were recruited from the same university to minimize sample heterogeneity. Female athletes were recruited from three varsity sports teams, and studied at the same time during the beginning (In-Season) and end of season (Off-Season). This study was approved by the University of Western Ontario's Health Sciences Research Ethics Board. Informed consent was obtained from each participant prior to the start of data collection. All participants in this study were university level athletes or students (age 18–30 years old). Athletes and sedentary recruits were required to be concussion free for at least 6 months to be included in the study. Six months was chosen because concussion symptom resolution typically occurs within a month, ensuring no athletes with chronic post-concussion syndrome were included. Athletes were recruited from the women's varsity rugby team over the course of five seasons, and women's varsity rowing and swim teams over a single season as describe previously (Schranz et al., 2018; Manning et al., 2020). Other exclusion criteria included any pre-existing systemic disease (e.g., diabetes, autoimmune diseases, immunosuppressed subjects), any history of head or eye injury involving metal fragments, and implanted electrical devices (e.g., cardiac pacemaker).

Briefly, sports seasons began at the end of August and early September (including tryouts). Rugby players had weekly contact practices in addition to weekly games until November, followed by non-contact training until off-season data collection. Rowers trained 6 days a week with regular regatta competitions until November, and swimmers trained 6 days a week with monthly swim meets until March. Athletes may have participated in other sports outside of their sports season, and therefore outside of this study (e.g., April through August). In-Season data were acquired from the end of August to early September for all athletes, and Off-Season data were acquired from the end of January through February for rugby players and rowers, and in March for swimmers. Sedentary participants were scanned at a single time point from the end of January to end of March, to match with the Off-Season timepoint in athletes.

### Clinical Measures

The SCAT3 (Sport Concussion Assessment Tool) was administered by a sports medicine physician at the In- and Off-Season timepoints for all athletes, and results are reported elsewhere (Manning et al., 2019).

For the sedentary group, participants were recruited using the IPAQ (International Physical Activity Questionnaire, October 2002), a self-administered questionnaire that incorporates (1) job-related physical activity, (2) transportation physical activity, (3) housework, house maintenance, and caring for family, and (4) recreation, sport, and leisure-time physical activity, where each section is comprised of walking, moderate, and vigorous activity levels. Participants were asked to fill out the questionnaire based on their average routine over the course of the school semester (September 2018–January 2019), to match with the duration of the varsity sports seasons. This required participants to record their average time spent per activity outlined in each of the four categories. The raw time spent per activity was then weighted by its energy requirements defined in METs (METabolic equivalent; ratio of work metabolic rate to a standard resting metabolic rate) to yield a score of MET-minutes. A MET-minute is computed by multiplying the MET score of an activity by the minutes performed. The MET score used for walking, moderate, and vigorous activities were 3.3, 4.0, and 8.0, respectively (Ainsworth et al., 2000). Additionally, for transportation by cycling a MET score of 6.0 was used (Ainsworth et al., 2000). The IPAQ categorizes participants, based on their MET score, into low (<600 MET minutes), moderate (at least 600 MET minutes), or high (at least 3000 MET minutes) activity levels. A typical week of training for athlete participants would place them in the high IPAQ group (a typical week of training alone for contact athletes would yield a MET score of ~5,000), and for that reason athletes did not complete an IPAQ form. Instead, the IPAQ was reserved as an inclusion criteria for the sedentary group with an arbitrary threshold of 3000 MET min (e.g., participants with low and moderate activity), since there is no well-accepted threshold for sedentary behaviors (Guidelines for Data Processing and Analysis of the International Physical Activity Questionnaire (IPAQ) - Short and Long Forms, 2005).

### Magnetic Resonance Imaging Acquisition

**Figure 1:**
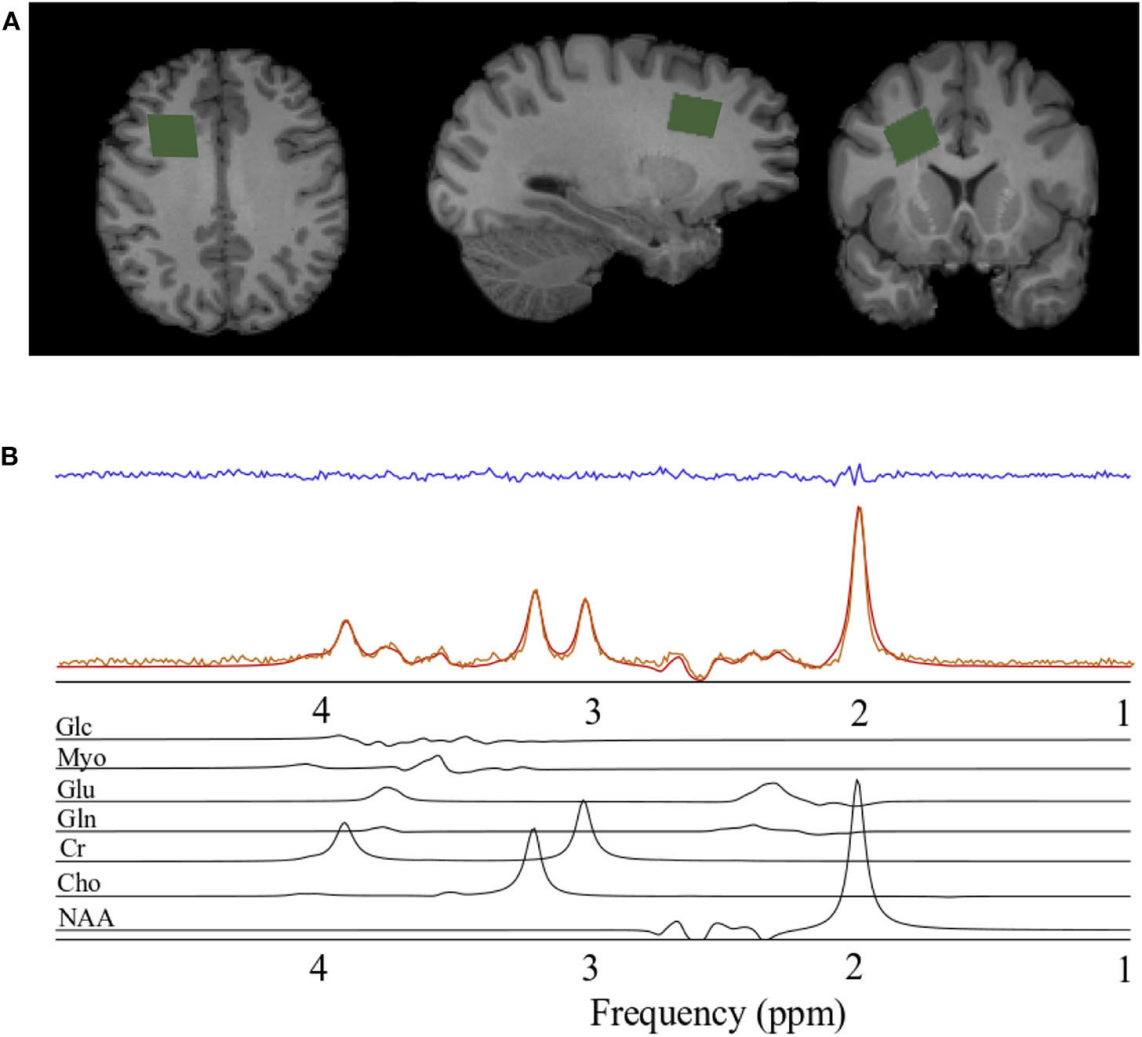
Spectroscopy voxel localization and spectral fitting**. (A)** From left to right; axial, sagittal, and coronal views of a T_1_-weighted anatomical image with the spectroscopy voxel overlaid in green in the right prefrontal region. **(B)** Spectrum acquired (orange) from the voxel in A, reconstructed spectrum (red), the residual after fitting (blue), and the individual prior knowledge components of the spectrum shown below in black. Glc, Glucose; Myo, *Myo*-inositol; Glu, Glutamate; Gln, Glutamine; Cr, Creatine; Cho, Choline; NAA, *N*-acetyl aspartate; ppm, parts per million.

The magnetic resonance spectroscopy data acquisition was identical to that reported previously (Schranz et al., 2018). Briefly, a Siemens 3T Magnetom Tim Trio and Prisma Fit MRI Scanners (Erlangen, Germany), both using a 32-channel head coil, were used for data acquisition. A rapid T_2_-weighted FLAIR image was acquired to guide the placement of a 6 cm^3^ (2 × 2 × 1.5 cm) voxel in the right prefrontal WM region of the brain (Figure 1A) for the acquisition of the spectroscopy data (slices = 50, TR/TE = 15,000/139 ms, slice thickness = 3 mm, turbo factor = 38, matrix size 256 × 256, FOV = 256 mm, inversion time = 2,850 ms). The right prefrontal white matter region was chosen to explore associations between metabolite levels and diffusion abnormalities previously described in this region following concussion and to obtain a sample of white matter that minimized contributions from gray matter and cerebral spinal fluid. Water suppressed (192 acquisitions) and unsuppressed (8 acquisitions) spectroscopy data were acquired using the PRESS (point resolved spectroscopy) pulse sequence (TR/TE = 2,000/135 ms, dwell time = 833 μs, number of points = 1,024). A long echo-time was chosen in the current study to reduce the metabolite measurement error associated with the macromolecule baseline at shorter echo times (Wong et al., 2018). Anatomical images for the estimation of voxel gray matter (GM), white matter (WM), and CSF volume were acquired using a sagittal T_1_-weighted magnetization-prepared rapid acquisition gradient echo sequence (TR/TE = 2,300/2.94 ms, flip angle = 98, matrix size 256 × 256, FOV = 256 × 240 mm, number of slices = 160, slice thickness = 1.22 mm).

### Magnetic Resonance Spectroscopy Analysis

Spectra were processed and analyzed as previously described Schranz et al. (2018), with absolute metabolite levels quantified using the approach described in Gasparovic et al. (2006). A signal to noise ratio (SNR) <50 or water line width >12 Hz were used as a quality control threshold for spectra. SNR was measured as the *N*-acetyl aspartate peak height divided by the standard deviation of the noise. Briefly, spectra were lineshape corrected by combined QUALITY (Quantification improvement by converting lineshapes to the lorentzian type) deconvolution and eddy current correction (Bartha et al., 2000) and fitted in the time domain using a Levenberg–Marquardt minimization routine (Bartha et al., 1999) using prior knowledge of metabolite line shapes (Figure 1B). Analysis software created in our laboratory in the IDL (version 5.4 Research Systems Inc., Boulder, CO) programming language was used to model the spectra using prior knowledge acquired from *in vitro* spectra obtained from aqueous solutions of metabolites at pH 7.0 prior to the study (Bartha et al., 1999). Absolute concentrations are reported using unsuppressed water from the MRS voxel as an internal standard as previously described (Bartha et al., 1999; Goncalves et al., 2016). This approach incorporates corrections to account for tissue partial volume (GM, WM, CSF) using the segmented T_1_-weighted anatomical image, as well as corrections for signal loss due to T_1_ and T_2_ relaxation. The relaxation time constants used in this study are provided in Table 1 (Wansapura et al., 1999; Mlynarik et al., 2001; Ethofer et al., 2003; Träber et al., 2004; Lu et al., 2005; Stanisz et al., 2005; Piechnik et al., 2009; Ganji et al., 2012; Harris et al., 2015; Zhang and Shen, 2016; Wyss et al., 2018). To eliminate the uncertainty associated with partial volume correction, and to compare to the literature, metabolite ratios relative to creatine (X/Cr) were also calculated. The reproducibility of voxel placement within subjects, between time points was assessed by calculating the relative fraction of GM, WM, and CSF in the voxels, and by registering the follow-up anatomical images to the baseline images to quantify the voxel overlap using the Dice index (Dice, 1945) in a subset of athletes.

**Table 1:** 3T relaxation constants used in absolute quantification.

**Metabolite**		**Gray matter**		**White matter**		**Cerebrospinal fluid**	
**# of Protons**		**T_**1**_ (s)**	**T_**2**_ (ms)**	**T_**1**_ (s)**	**T_**2**_ (ms)**	**T_**1**_ (s)**	**T_**2**_ (ms)**
*N*-acetyl aspartate	3	1.34	318	1.35	343	-	-
Choline	9	1.21	246	1.26	209	-	-
Creatine	3	1.34	158	1.36	159	-	-
Glutamine	5	1.17	134	0.98	134	-	-
Glutamate	5	1.27	167	1.17	143	-	-
*Myo*-inositol	6	1.17	221	0.98	195	-	-
Glucose	6	1.17	117	0.98	122	-	-
Water	2	1.46	95	0.94	75	4.3	503

*T_1_, Longitudinal relaxation; T_2_, Transverse relaxation*.

*All values used were averaged from values reported in the literature: Wansapura et al. (1999); Mlynarik et al. (2001); Ethofer et al. (2003); Träber et al. (2004); Lu et al. (2005); Stanisz et al. (2005); Piechnik et al. (2009); Ganji et al. (2012); Harris et al. (2015); Zhang and Shen (2016); Wyss et al. (2018)*.

### Statistical Analysis

All statistical analyses were performed using GraphPad Prism Version 8.0 for Mac OS X (GraphPad Software, San Diego, CA). For each metabolite, the ROUT method (maximum desired false discovery rate Q = 1%, within groups) was performed to remove outliers and the D'Agostino & Pearson normality test was used to test for normality. The sequence of statistical comparisons performed in this study are summarized in Table 3. First, a one-way ANOVA, followed by two-tailed Tukey's multiple comparisons test, was used to test for differences across Sedentary and Athletic Groups (Rowers, Swimmers, and Rugby Players). The Kruskal-Wallis, followed by Dunn's multiple comparisons test, was used for metabolites that did not pass the normality test.

Before combining rowers and swimmers into the “Non-Contact” group, a two-way repeated measures ANOVA, followed by two-tailed Sidak's multiple comparisons tests, was used to test for differences between the rowers and swimmers at both measurement times. Then, a two-way repeated measures ANOVA was used to test for differences between non-contact (rowers and swimmers) and contact (rugby) athletes, at In- and Off-Season time points. In both cases, the repeated measures were within groups across time (In- and Off-Season). A *p*-value of 0.008 (Bonferroni corrected for 6 metabolite comparisons) was used for all statistical tests, and all multiple comparisons were corrected using the relevant method (e.g., for a one-way ANOVA, multiple comparisons are corrected using Tukey, while for Kruskal-Wallis, multiple comparisons are corrected using Dunn's), such that a *p*-value of 0.05 was used.

## Results

### Participants, Clinical Data, and Concussion History

Twenty-five participants were recruited to the sedentary group. Two participants were eliminated because one suffered a head injury prior to their scan, and another had a total MET-minutes >3000, leaving a total of *N* = 23 participants in the sedentary group. On average, participants had mean (± standard deviation) MET-minutes of 1252 ± 952. Of the 23 included participants (Age 24 ± 2.8), six reported having a previous concussion, 9.3 ± 5.7 years prior to the start of the study.

A total of 31 athletes were recruited into the non-contact group from the Western varsity rowing and swim teams, with a total of 23 completing both scans at the In- and Off-Season time points (Rowers, *n* = 10, Age = 20±1.98; Swimmers *n* = 13, Age = 19.2 ± 1.5). Of these 23 athletes, three reported having a previous concussion prior to the beginning of the study (Rowers *n* = 2; Swimmers *n* = 1) Athletes were not asked specifically how long it had been since any previous concussions, but only if previous concussions had occurred more than 6 months prior to study enrollment. For details on the 54 rugby athletes included in comparisons and associated concussion histories, please see Schranz et al. (2018). Briefly, of the 54 rugby athletes, 14 played two consecutive seasons, 9 played three consecutive seasons, and one played two seasons, interspersed with a season of non-play. Additionally, the results of the SCAT3 scores for the contact and non-contact teams are reported in Manning et al. (2020) and Schranz et al. (2018).

### Quality Assurance Measures

All spectroscopy quality assurance measures calculated for the sedentary and non-contact groups did not differ significantly from the measures previously reported in the contact athletes (Schranz et al., 2018). The spectroscopy voxel was placed in the right prefrontal region with mean (± standard deviation) tissue content in the sedentary group: GM 23 ± 6%, WM 73± 8%, CSF 3.5 ± 2%, and non-contact group: GM 23 ± 7%, WM 74 ± 8%, CSF 2.7 ± 2%. As previously reported by Schranz et al. (2018), the voxel overlap between In-Season and Off-Season scans in the contact group was 46% on average using the Dice similarity coefficient. For all spectra from the sedentary and non-contact groups, the average full-width at half maximum of the water peak was 6.2 Hz and the average SNR was 92. All spectra and residuals were visually inspected prior to statistical analyses for artifacts and no spectra were eliminated from the analysis due to artifacts or insufficient quality (SNR <50 or water linewidth >12 Hz). Cramer-Rao Lower Bounds (CRLB) were calculated for all metabolites but not used to eliminate spectra to avoid selection bias (Kreis, 2016). For all spectra (n=184), the average CRLBs were 0.62% for *N*-acetyl aspartate, 1.5% for choline, 1.3% for creatine, 56% for glutamine, 4.2% for glutamate, and 6.9% for *myo*-inositol. Outliers were identified using the ROUT method, leading to the removal of two data sets in the Contact Group when evaluating glutamate and creatine (*N* = 52), and the removal of six data sets from the Contact Group when evaluating *myo*-inositol (*N* = 48).

### Magnetic Resonance Spectroscopy Absolute Concentrations

#### One-Way ANOVA: Sedentary, Rowers, Swimmers, Rugby

**Figure 2:**
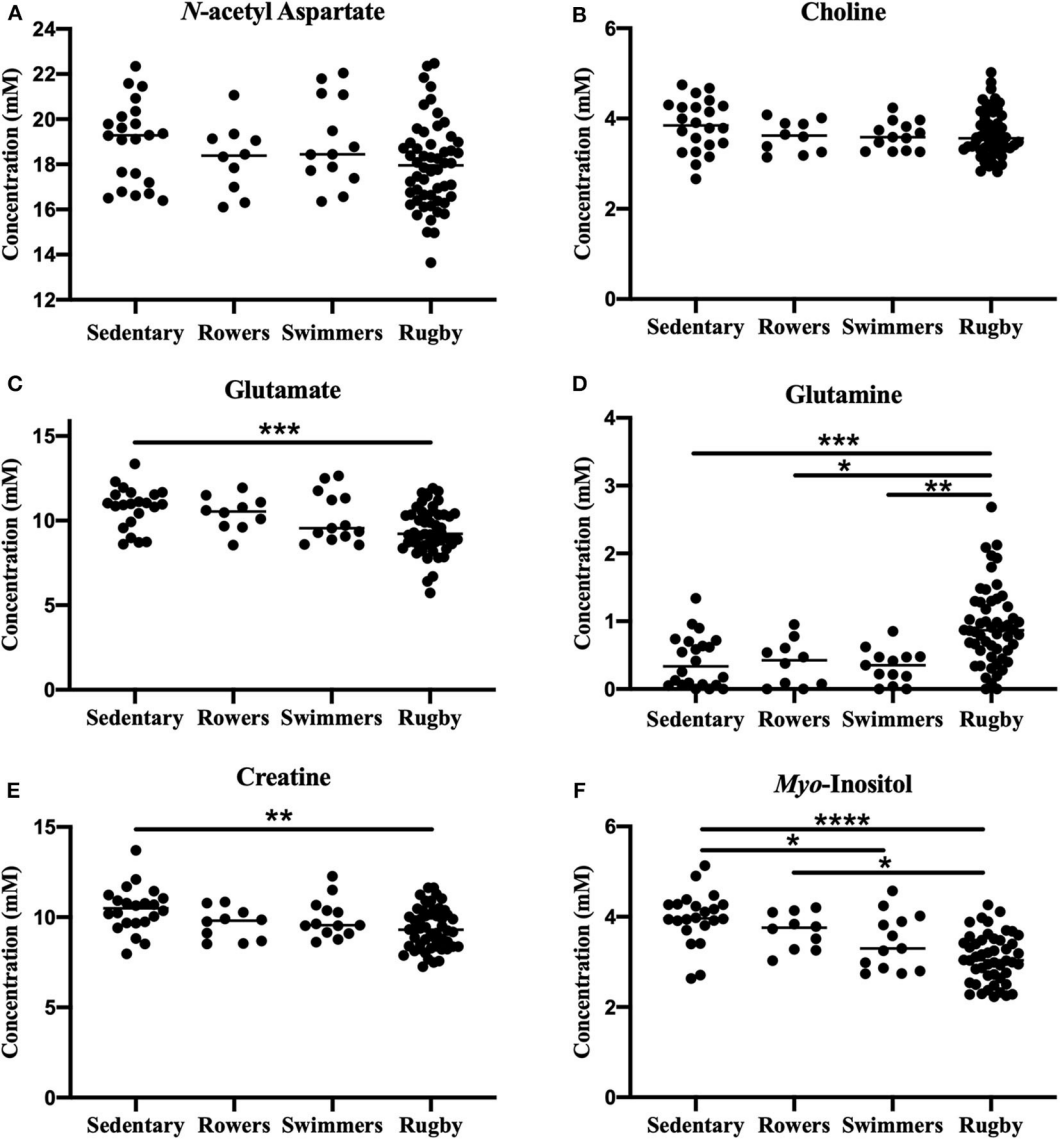
Off-Season Athletes compared to a sedentary group: Measured concentration for **(A)** *N*-acetyl aspartate, **(B)** choline, **(C)** glutamate, **(D)** glutamine, **(E)** creatine, and **(F)** *myo*-inositol across groups. Error bars represent the standard error of the mean *(p* < 0.0001****; p < 0.001***; p < 0.01**; p < 0.05*).

A one-way ANOVA was used to compare the Sedentary females to Rowers, Swimmers, and Rugby athletes at their Off-Season. A Kruskal-Wallis Test was used instead of an ANOVA for glutamate and glutamine. The ANOVA revealed no significant differences among the four groups in *N*-acetyl aspartate [Figure 2A; *F*_(3, 95)_ = 2.24, *p* = 0.088] or choline [Figure 2B; *F*_(3, 95)_ = 2.02, *p* = 0.116]. However, significant differences were found in glutamate (Figure 2C; Kruskal-Wallis statistic = 15.67, *p* = 0.0013), glutamine (Figure 2D; Kruskal-Wallis statistic = 26.06, *p* < 0.0001), creatine [Figure 2E; *F*_(3, 94)_ = 4.71, *p* = 0.0042], and *myo*-inositol [Figure 2F; *F*_(3, 89)_ = 14.39, *p* < 0.0001] levels. More specifically, Rugby athletes had significantly lower glutamate (Dunn's multiple comparisons test, *p* = 0.0014) and significantly lower creatine levels (Tukey's multiple comparison test, *p* = 0.0021) than the sedentary group, and significantly higher glutamine levels than Sedentary (Dunn's, *p* = 0.0009), Rowers (Dunn's, *p* = 0.02), and Swimmers (Dunn's, *p* = 0.0012). Additionally, Rugby athletes had significantly lower *Myo*-inositol levels compared to the Sedentary group (Tukey, *p* < 0.0001) and Rowers (Tukey, *p* = 0.011). Finally, Swimmers had significantly lower *myo*-inositol levels compared to the Sedentary group (Tukey, *p* = 0.034).

#### One-Way ANOVA: Sedentary, Non-contact, Contact

**Figure 3:**
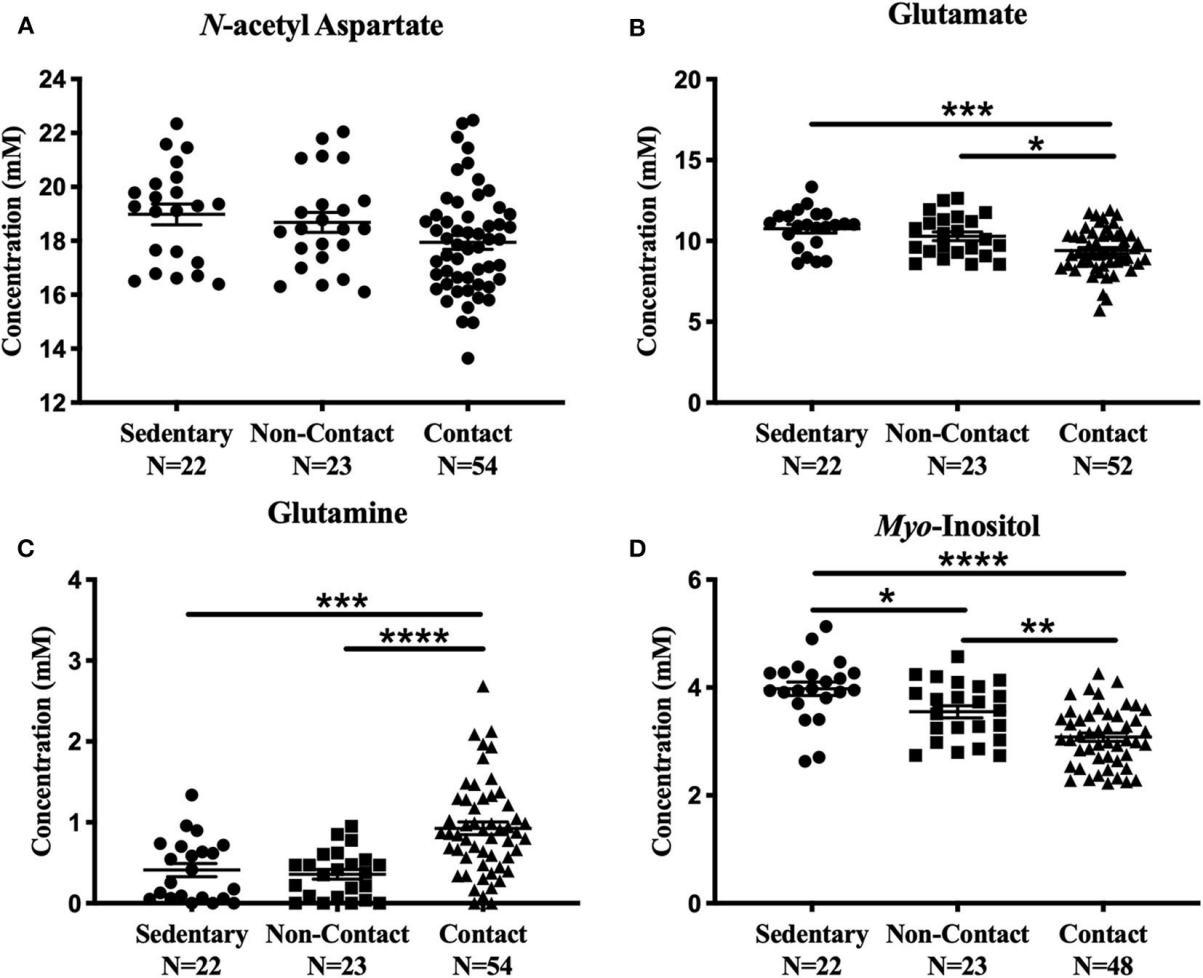
Off-Season Non-Contact, Contact compared to Sedentary Control: Measured concentration for **(A)** *N*-acetyl aspartate, **(B)** glutamate, **(C)** glutamine, and **(D)** *myo*-inositol across groups. Error bars represent the standard error of the mean *(p* < 0.0001****; p < 0.001***; p < 0.01**; p < 0.05*).

No significant metabolite level differences were found between the rowers and swimmers at either time point, so these athletes were combined into a single non-contact athlete group. Comparing metabolite levels between these three groups, the findings were similar to those described above for the four*-*group comparison, and are provided in Figure 3 for completeness. No significant differences were found among the three groups in *N*-acetyl aspartate [Figure 3A; *F*_(2, 96)_ = 2.91, *p* = 0.059] or choline [Fig not shown; *F*_(2, 96)_ = 1.43, *p* = 0.24]. However, glutamate [Figure 3B; ANOVA, *F*_(2, 94)_ = 9.39, *p* = 0.0002], glutamine [Figure 3C; ANOVA, *F*_(2, 96)_ = 15.5, *p* < 0.0001], creatine [Fig not shown; ANOVA, *F*_(2, 95)_ = 6.92, *p* = 0.0016], and *myo*-inositol [Figure 3D; ANOVA, *F*_(2, 90)_ = 21.02, *p* < 0.0001] were significantly different. No differences were found between the sedentary group and non-contact group, except for lower *myo*-inositol levels (Figure 3D; Tukey's, q_90_ = 3.69, *p* = 0.028) in the non-contact group.

#### Two-Way ANOVA: Non-contact and Contact Over a Season of Play

**Figure 4:**
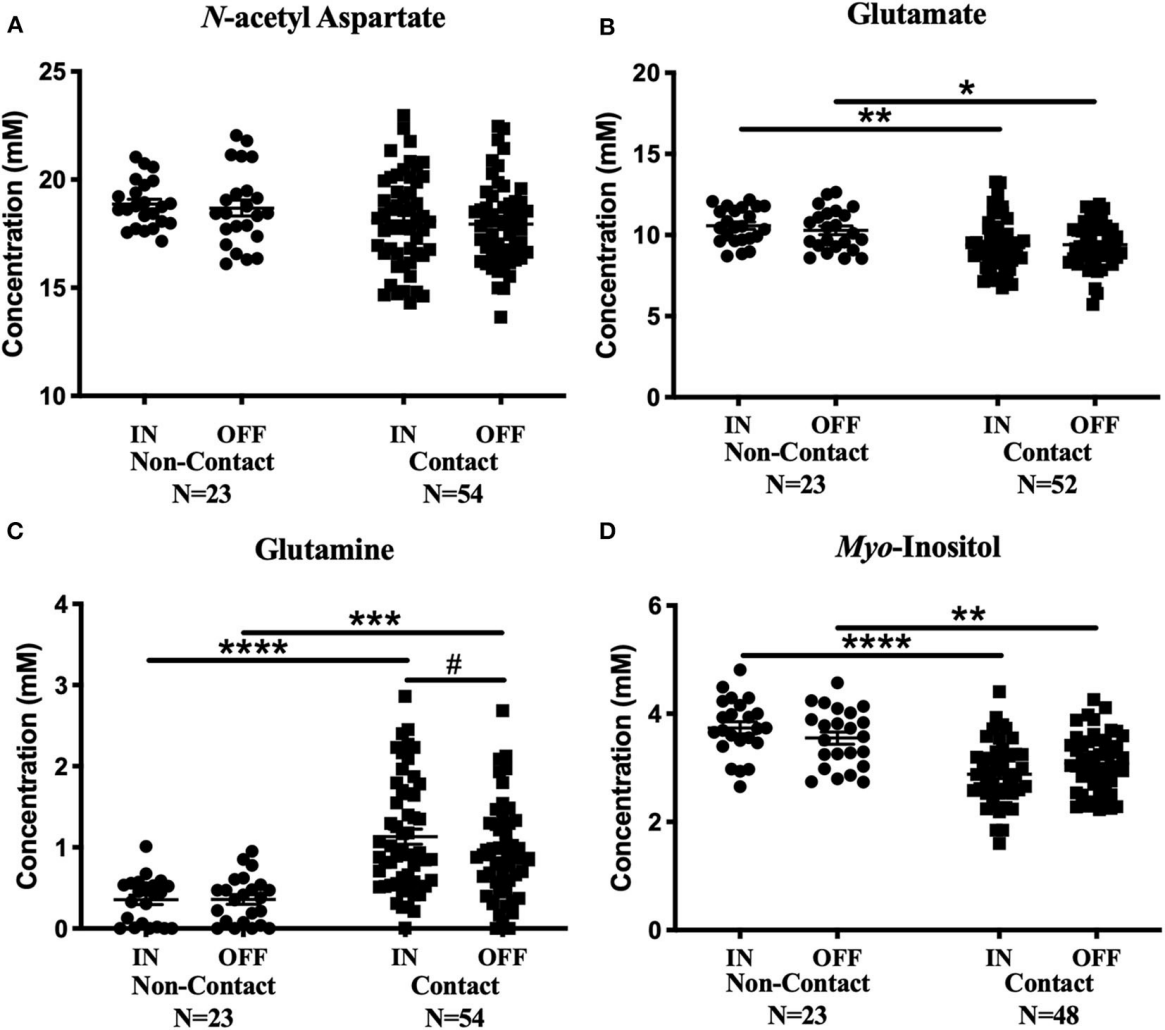
Contact vs. Non-contact athlete's metabolite concentrations: Measured concentration for **(A)** *N*-acetyl aspartate, **(B)** glutamate, **(C)** glutamine, and **(D)** *myo*-inositol in the non-contact and contact group. Error bars represent the standard error of the mean. ^#^Represents the decrease in glutamine levels previously reported (Schranz et al., 2018). *(p* < 0.0001****; p < 0.001***; p < 0.01**; p < 0.05*).

No metabolite changes were detected over a season of play in the non-contact group (Figure 4). There were no significant differences between contact and non-contact groups in levels of *N*-acetyl aspartate [Figure 4A; Two-way ANOVA, *F*_(1, 75)_ = 3.63, *p* = 0.06], choline [Two-way ANOVA, *F*_(1, 75)_ = 0.99, *p* = 0.32, data not shown], or creatine [Two-way ANOVA, *F*_(1, 74)_ = 1.9, *p* = 0.17, data not shown].

Mean glutamate concentrations were found to be significantly different between contact and non-contact groups [Figure 4B; Two-way ANOVA, *F*_(1, 73)_ = 11.67, *p* = 0.001]. Glutamate was lower in the contact group at the In-Season (Sidak's, *t*_146_ = 3.36, *p* = 0.002) and Off-Season (Sidak's*, t*_146_ = 2.53 *p* = 0.025) time points, although glutamate levels did not change between time points within either group. Additionally, mean glutamine concentrations were also found to be significantly different between contact and non-contact groups [Figure 4C; Two-way ANOVA, *F*_(1, 75)_ = 33.06, *p* < 0.0001], with higher concentrations in the contact group at the In-Season (Sidak's, *t*_150_ = 5.6, *p* < 0.0001) and Off-Season (Sidak's, *t*_150_ = 4.1, *p* = 0.0001) time points.

Mean *myo*-inositol concentrations were found to be significantly different between contact and non-contact groups [Figure 4D; Two-way ANOVA, *F*_(1, 69)_ = 36.78, *p* < 0.0001], with lower concentrations in the contact group at the In-Season (Sidak's, *t*_138_ = 6.14, *p* < 0.0001) and Off-Season (Sidak's, *t*_138_ = 3.36, *p* = 0.002) time points.

### Magnetic Resonance Spectroscopy Metabolite Ratios

#### One-Way ANOVA: Sedentary, Rowers, Swimmers, Rugby

A one-way ANOVA was used to compare the Sedentary females to Rowers, Swimmers, and Rugby athletes at their Off-Season. A Kruskal-Wallis Test was used instead of an ANOVA for *N*-acetyl aspartate/Cr, Choline/Cr, Glutamate/Cr and Glutamine/Cr, and the results can be found in Table 2. No significant differences among the four groups were found in *N*-acetyl aspartate/Cr (Kruskal-Wallis statistic = 5.04, *p* = 0.169), Choline/Cr (Kruskal-Wallis statistic = 9.64, *p* = 0.022), or Glutamate/Cr (Kruskal-Wallis statistic = 7.76, *p* = 0.051). Significant differences were found in Glutamine/Cr (Kruskal-Wallis statistic = 29.51, *p* < 0.0001) and *Myo*-inositol/Cr [Table 2; *F*_(3, 93)_ = 8.41, *p* < 0.0001] levels. More specifically, Rugby athletes had significantly higher Glutamine/Cr ratios than Sedentary (Dunn's, *p* = 0.0002), Rowers (Dunn's, *p* = 0.011), and Swimmers (Dunn's, *p* = 0.0007). Finally, Rugby athletes had significantly lower *Myo*-inositol/Cr compared to the Sedentary group (Tukey, *p* = 0.0002) and Rowers (Tukey, *p* = 0.006).

**Table 2:** Off-Season mean metabolite ratios to creatine.

	**Off-Season metabolite ratio means** **±** **standard deviation**				
	**Sedentary**	**Rowers**	**Swimmers**	**Non-contact**	**Contact**
*N*-acetyl aspartate/Creatine	1.6 ± 0.10	1.7 ± 0.15	1.7 ± 0.12	1.7 ± 0.13	1.7 ± 0.14
Choline/Creatine	0.75 ± 0.076	0.77 ± 0.068	0.73 ± 0.052	0.76 ± 0.069	0.79 ± 0.077
Glutamate/Creatine	0.69 ± 0.054	0.73 ± 0.045	0.68 ± 0.040	0.70 ± 0.050	0.67 ± 0.0096
Glutamine/Creatine	**0.024** **±** 0.022^****^	**0.023** **±** 0.021^*^	**0.021** **±** 0.017^***^	**0.022** **±** 0.018^****^	0.061 ± 0.036
*Myo*-inositol/Creatine	**0.41** **±** 0.041^***^	**0.41** **±** 0.010^**^	0.37 ± 0.016	**0.39** **±** 0.052^*^	0.35 ± 0.050

*Significant compared to the Contact Group*:

* p < 0.05;

** p < 0.01;

*** p < 0.001;

**** *p < 0.0001. Bold values indicate statistical significance*.

#### One-Way ANOVA: Sedentary, Non-contact, Contact

A one-way ANOVA revealed no significant differences among the three groups in *N*-acetyl aspartate/Cr [*F*_(2, 95)_ = 2.82, *p* = 0.065], Choline/Cr [*F*_(2, 95)_ = 2.94, *p* = 0.058], or Glutamate/Cr [*F*_(2, 95)_ = 2.099, *p* = 0.13]. However, significant differences were found among Glutamine/Cr [*F*_(2, 95)_ = 19.06, *p* < 0.0001] and *Myo*-inositol/Cr [*F*_(2, 94)_ = 10.29, *p* < 0.0001].

The contact group had significantly elevated Glutamine/Cr compared to non-contact (Tukey's, q_95_ = 7.23, *p* < 0.0001) and sedentary groups (Tukey's, q_95_ = 6.77, *p* < 0.0001), while *Myo*-inositol/Cr was significantly lower in the contact group compared to the non-contact (Tukey's, q_94_ = 3.84, *p* = 0.021) and sedentary groups (Tukey's, q_94_ = 6.04, *p* = 0.0001). No differences were found between the sedentary group and non-contact group.

#### Two-Way ANOVA: Non-contact and Contact Over a Season of Play

No metabolite ratio changes were found over a season of play in the non-contact group (Table 3). A two-way ANOVA found no significant differences between contact and non-contact groups in *N*-acetyl aspartate/Cr [*F*_(1, 74)_ = 0.059, *p* = 0.8], while mean Choline/Cr was significantly different between athlete groups [*F*_(1, 73)_ = 10.05, *p* = 0.0022], with significantly higher Choline/Cr in the contact group at the In-Season time point (Sidak's, *t*_146_ = 3.38, *p* = 0.0019). Mean Glutamate/Cr was found to be significantly different between contact and non-contact groups [Table 3; *F*_(1, 74)_ = 10.7, *p* = 0.0016], with a lower ratio in the contact group at the In-Season (Sidak's, *t*_148_ = 3.43, *p* = 0.0016). Additionally, mean Glutamine/Cr were found to be significantly different between contact and non-contact groups (Table 3; *F*_(1, 74)_ = 41.87, *p* < 0.0001), with a higher ratio in the contact group at the In-Season (Sidak's, *t*_148_ = 6.18, *p* < 0.0001) and Off-Season (Sidak's, *t*_148_ = 4.45, *p* < 0.0001). Mean *Myo*-inositol/Cr were found to be significantly different between contact and non-contact groups [Table 3; *F*_(1, 71)_ = 14.19, *p* = 0.0003], with lower concentrations in the contact group at the In-Season (Sidak's, *t*_142_ = 3.82, *p* = 0.0004).

**Table 3:** Mean metabolite ratios non-contact vs. contact.

	**Metabolite ratio non-contact vs. contact means** **±** **standard deviation**			
	**Non-contact**		**Contact**	
	**In-Season**	**Off-Season**	**In-Season**	**Off-Season**
*N*-acetyl aspartate/Creatine	1.7 ± 0.12	1.7 ± 0.13	1.7 ± 0.14	1.69 ± 0.14
Choline/Creatine	**0.74** **±** 0.052^**^	0.75 ± 0.065	0.81 ± 0.087	0.79 ± 0.077
Glutamate/Creatine	**0.72** ±0.040^**^	0.70 ± 0.048	0.66 ± 0.077	0.67 ± 0.070
Glutamine/Creatine	**0.020** ±0.015^****^	**0.022** **±** **0.018**^**†**^	0.075 ± 0.043	**0.061** **±** **0.036**^**#**^
*Myo*-inositol/Creatine	**0.41** **±** 0.053^****^	0.39 ± 0.052	0.35 ± 0.078	0.36 ± 0.050

Significant compared to the Contact In-Season Group:

** p < 0.01;

**** p < 0.0001.

Significant compared to the Contact Off-Season Group:

† *p < 0.0001*.

# *The drop in Glutamine/Creatine previously reported Schranz et al. (2018)*.

*Bold values indicate statistical significance*.

## Discussion

The objective of this study was to compare brain metabolite levels between female varsity athletes engaged in contact and non-contact sports to examine the long-term effects of physical contact, while accounting for exercise effects with a sedentary group. Brain metabolite levels were acquired from a single voxel in the right prefrontal white matter. We found that Rowers and Swimmers exhibited a small reduction in *myo*-inositol levels, but had an otherwise similar metabolite profile to sedentary individuals. In contrast, Rugby players exhibited a different metabolite profile than sedentary individuals consisting of lower *myo*-inositol, glutamate, and creatine, and higher glutamine levels. When comparing changes during and after the sports season, *myo*-inositol and glutamate were found to be lower and glutamine was found to be higher in contact (Rugby) athletes compared to non-contact (Rowers and Swimmers) athletes. The observed metabolite differences between groups and across time highlight the importance of using appropriate control groups when studying changes in the brain following concussion in sport.

### Non-contact Athletes vs. Sedentary

A small reduction in *myo*-inositol was observed in the prefrontal white matter in non-contact athletes compared to sedentary individuals. No other metabolite differences were found. Moreover, no significant changes were measured from In- to Off-Season in the non-contact athletes. Previous studies have investigated the acute effects of physical activity, and have found increased lactate and Glx, as well as acute modulation of glutamate and GABA after vigorous exercise (Maddock et al., 2011, 2016). Additionally, Gonzales et al. (2013) found elevated *N*-acetyl aspartate/Cr and Choline/Cr in an endurance trained group (>40 years of age) compared to normal healthy controls. These differences were not observed in the current study suggesting that the acute changes in brain metabolite levels previously reported do not translate into a chronic adaptation in the metabolite profile measured by MRS.

### Contact Athletes vs. Sedentary

There were several metabolite level differences between the contact (Rugby) group and the sedentary group consisting of lower *myo*-inositol, glutamate, and creatine, and higher glutamine levels. A previous study that examined high school football players found metabolite differences at the beginning of season, with lower choline and creatine levels during the season in the motor cortex and lower creatine in the dorsolateral prefrontal cortex (Poole et al., 2014). These results are consistent with the lower creatine levels we observed at the off-season in Rugby players compared to our sedentary group. Moreover, similar to the elevated glutamine levels in contact athletes observed in the current study, a study by Bari et al. (2018) examining male football and female soccer players found elevated Glx (glutamate + glutamine) levels in the motor cortex in females. Furthermore, high school football players were found to have elevated Glx in a similar region of interest at the beginning of a sports season, and these Glx levels were found to drop during the season. Interestingly, this change in Glx follows the same pattern as glutamine in our contact athletes. Other studies have reported reduced *N*-acetyl aspartate in male mixed martial arts fighters (Mayer et al., 2015), and in female and male athletes without concussion (Chamard et al., 2012, 2014; Panchal et al., 2018). Moreover, Chamard et al., 2012 found reduced *N*-acetyl aspartate/Cr in the corpus callosum of female athletes only, but with subsequent absolute quantification, found *N*-acetyl aspartate to be reduced in males and females (Panchal et al., 2018). In the current study, no significant changes in *N*-acetyl aspartate were observed, however this may be due to the different voxel positions between studies. Since *N*-acetyl aspartate is constantly transported along axons, it may be more feasible to observe subtle changes in *N*-acetyl aspartate in a larger volume of WM, such as the corpus callosum, rather than our current voxel position. Alternatively, it is conceivable that metabolite changes are regionally dependent across the brain. For example, in this current study we also did not observe significant changes in creatine or choline, suggesting that the prefrontal WM is not in an energy crisis nor is there change in membrane turn-over rates.

No metabolite changes were found between the In and Off-Season in contact athletes, except for a decrease in glutamine levels, as previously reported (Schranz et al., 2018). This difference found only in the contact athletes suggests that the contact nature of the sport is having a cumulative effect on the brain, as described by others (Koerte et al., 2012; Mayer et al., 2015; Lefebvre et al., 2018). To confirm that the contact athletes were indeed receiving high impacts, a head impact sensor that measures linear acceleration and rotational velocity was worn by a subset of players during a preseason rugby game (Manning et al., 2020). During this single rugby game, a total of 151 impacts exceeding 15 g were recorded across 26 players. Therefore, on average, a single player received six impacts >15 g per practice, and two impacts >15 g per game. Detailed results on the impact data can be found in Manning et al. (2020). It is important to recognize that previous studies reporting higher impact rates used a threshold of >10 g (King et al., 2015, 2018), rather than the >15 g used in this cohort of athletes. Additionally, it has been shown that 45% of head impacts land between 10 and 15 g (King et al., 2016), meaning that there is a large portion of impacts not included in the current analysis. These *sub-concussive* impacts experienced throughout the season may contribute to the reduction in glutamine and Glutamine/Cr, as well as to the other metabolite level differences observed in the female rugby athletes compared to the sedentary and non-contact groups as discussed in the next section.

### Non-contact vs. Contact Athletes

There were several metabolite level differences between the contact (Rugby) and non-contact (Swimmer and Rower) groups. For example, the current study found lower *myo*-inositol levels at In- and Off-Season in the prefrontal white matter of contact athletes in this study. This is different from a recent study by Lefebvre et al. (2018) examining male and female athletes, which found increased *myo*-inositol in contact athletes (rugby and soccer), compared to non-contact (swimming) and non-athletes in the motor cortex. Furthermore, studies of retired contact athletes have found higher *myo*-inositol levels in the medial temporal lobe (Tremblay et al., 2013) and in the posterior cingulate gyrus (Koerte et al., 2015). Differences in voxel position and the age of study participants may explain these differences.

Although the accumulation of sub-concussive impacts throughout the sports season in contact athletes may contribute to the observed metabolite level differences between groups, we must also consider the type of exercise utilized by these athletes. During exercise there are three main types of energy systems that are used to generate energy (ATP) for force generation. These are the phosphagen and glycolytic systems (anaerobic metabolism), and the oxidative system (aerobic metabolism) (Haff and Triplett, 2015). All three types are involved in exercise, but the extent of involvement of each system depends upon the type and duration of the activity or sport. For example, all athletes in the current study moderately used the glycolytic system, but rugby players primarily use the anaerobic phosphagen system, while rowers primarily use the aerobic oxidative system (Haff and Triplett, 2015). Swimming is dependent on the distance, where short distances rely on the anaerobic phosphagen system, and long distances rely on the aerobic system (Haff and Triplett, 2015). Unfortunately, it is difficult to tease apart the effect of repetitive sub-concussive impacts and the effect of exercise type because most contact sports primarily use anaerobic systems (Haff and Triplett, 2015). However, some non-contact athletes also primarily use anaerobic systems including short distance swimmers (included in the current study), track athletes, and powerlifters (Haff and Triplett, 2015). Since the metabolite profile of the swimmers in the current study did not differ from the rowers, we can conclude that the white matter metabolite profile is not modulated by the use of anaerobic compared to aerobic systems. Furthermore, this result suggests that differences observed within the contact group cannot be attributed to the type of exercise.

The reason for the altered metabolite profile in athletes involved in contact sports is unknown. We speculate that epigenetic changes in contact athletes may lead to altered enzyme expression, for example increased glutamine synthetase expression could lead to elevated glutamine levels. Practically, these changes require the use of control athletes participating in the same sport to study the effects of concussion.

### Reporting Glutamine

Most studies do not report glutamine separately from glutamate, but instead report these metabolites together as Glx. However, when these metabolites are combined, specificity is lost. In the current study, glutamine was quantified separately from glutamate despite the high Cramer-Rao lower bound of glutamine to better understand the role of both glutamine and glutamate in adaptation to sport. Due to the method used for data acquisition, the uncertainty in the glutamine measurement (CLRB) was high. However, CLRBs were not significantly different between groups (One-way ANOVA; *F* = 1.6, *p* = 0.21). Additionally, a significant difference was observed between the groups (e.g., Non-Contact and Contact athletes) due to a large effect size (Cohen's *d* = 1.25), where the contact athletes had mean glutamine levels that were 2.6 times greater than the non-contact athletes (e.g., 260% increase in mean levels).

Although glutamate and glutamine are tightly regulated as part of the glutamate-glutamine cycle, glutamine performs other roles in the brain, including its use as an energy substrate in microglia (Bernier et al., 2020). Moreover, changes in glutamate and glutamine are not inversely correlated, as would be expected if the role of glutamine was limited to its use within the glutamate-glutamine cycle to produce glutamate for neurotransmission. Furthermore, the current study highlights the need to develop spectroscopy techniques designed to increase sensitivity to low amplitude metabolites such as glutamine. These low amplitude metabolites are difficult to measure, and therefore often excluded from analyses, but may be critical to better understanding the cascade of metabolic events occurring in contact sport and post-concussion.

### Limitations, Strengths, and Future Work

There are several limitations to consider for this study. First, the MRS voxel was manually placed by the MRI technician, which limits the reproducibility of the voxel placement within subjects at follow up scans. However, given the distributed nature of concussive injury, and the heterogeneity of sub-concussive hits, it is likely that white matter damage is diffuse and extends well beyond the voxel. Second, data regarding the magnitude and severity of head impacts was not acquired in athletes throughout their seasons. Incorporating impact data could have been used to investigate associations between the number of contacts in the rugby players and metabolite levels. However, such data are difficult to acquire because the league did not allow impact recording devices to be worn during games. Third, some participants from the non-contact and sedentary groups did have a prior concussion history. However, concussions were few and all had resolved without lingering symptoms. Therefore, it is unlikely that these had a major effect on our results. However, it is worth noting that some studies have demonstrated long-term effects in people with a history of concussion (Martini and Broglio, 2018). In addition, all three groups in this study included individuals with a prior concussion history, therefore observed differences between the groups are likely due to other causes. Finally, the sports included in the current study had different cardiovascular demands such as maximal oxygen consumption (VO_2_ max) on athletes, and incorporated different types of exercise (aerobic vs. anaerobic). The effects of these differences on white matter metabolite profile are currently unknown. Future studies investigating these factors and their effects on the brain are warranted.

There are also several important strengths to the current study. First, the contact and non-contact athletes were recruited from three sports teams, ensuring similar training schedules and matched activity levels. Second, this study included only female athletes, as sex differences have been observed (Henry et al., 2011; Chamard et al., 2013), and studies in female athletes are underrepresented in the literature. Third, we chose to study the prefrontal white matter bordering the cortex since past studies have shown changes in this region following concussion (Henry et al., 2010; Poole et al., 2014), and it is known to be susceptible to damage due to shear forces during mild acceleration (Bayly et al., 2005). Finally, we chose a longer echo time (TE = 135 ms) for the spectroscopy acquisition to reduce the macromolecule signal contribution to the spectrum. Although a long echo-time does decrease spectral signal to noise ratio due to T_2_ relaxation, the elimination of the macromolecule baseline combined with metabolite modeling can increase quantification precision for many metabolites of interest.

## Conclusions

Metabolite levels in female varsity non-contact swimmers and rowers did not differ from age matched sedentary women except for a slightly lower *myo*-inositol level in the non-contact athletes. In contrast, female varsity rugby players had lower *myo*-inositol and glutamate, and higher glutamine levels compared to sedentary women and non-contact athletes. Importantly, glutamine levels were significantly higher in the athletes engaged in contact sport compared to the sedentary and non-contact groups. When examining the effect of concussion in sport, these data highlight the importance of using proper control groups to properly interpret the consequences of injury in the brain. Importantly, these results suggest that repetitive impacts due to physical contact in high impact sports can alter white matter metabolite levels.

## Data Availability Statement

The raw numerical data supporting the conclusions of this article will be made available by the authors, without undue reservation.

## Ethics Statement

The studies involving human participants were reviewed and approved by the University of Western Ontario's Health Sciences Research Ethics Board. The patients/participants provided their written informed consent to participate in this study.

## Author Contributions

AS wrote the manuscript. RB, GD, RM, and DF edited the manuscript. GD, LF, TD, DF, AB, JH, RM, and RB were involved in conceptualization and methodology of the study. CB and KB contributed to the formal analysis. All authors contributed to the article and approved the submitted version.

## Conflict of Interest

The authors declare that the research was conducted in the absence of any commercial or financial relationships that could be construed as a potential conflict of interest.

## References

[B1] Ainsworth B. E. Haskell W. L. Whitt M. C. Irwin M. L. Swartz A. M. Strath S. J.. (2000). Compendium of physical activities: an update of activity codes and MET intensities. Med Sci Sports Exerc. 32 (Suppl.), S498–S504. 10.1097/00005768-200009001-00009 10993420

[B2] Bari S. Svaldi D. O. Jang I. Shenk T. E. Poole V. N. Lee T.. (2018). Dependence on subconcussive impacts of brain metabolism in collision sport athletes: an MR spectroscopic study. Brain Imaging Behav. 13, 735–749. 10.1007/s11682-018-9861-9 29802602

[B3] Bartha R. Drost D. J. Menon R. S. Williamson P. C. (2000). Spectroscopic lineshape correction by QUECC: combined QUALITY deconvolution and eddy current correction. Magnetic Resonance Med. 44, 641–645. 10.1002/1522-2594(200010)44:4<641::AID-MRM19>3.0.CO;2-G 11025521

[B4] Bartha R. Drost D. J. Williamson P. C. (1999). Factors affecting the quantification of short echo *in-vivo* 1H MR spectra: prior knowledge, peak elimination, and filtering. NMR Biomed. 12, 205–216. 10.1002/(SICI)1099-1492(199906)12:4<205::AID-NBM558>3.0.CO;2-1 10421912

[B5] Bayly P. V. Cohen T. S. Leister E. P. Ajo D. Leuthardt E. C. Genin G. M. (2005). Deformation of the human brain induced by mild acceleration. J. Neurotrauma 22, 845–856. 10.1089/neu.2005.22.845 16083352 PMC2377024

[B6] Bernier L. P. York E. M. Kamyabi A. Choi H. B. Weilinger N. L. MacVicar B. A. (2020). Microglial metabolic flexibility supports immune surveillance of the brain parenchyma. Nat. Commun. 11:1559. 10.1038/s41467-020-15267-z 32214088 PMC7096448

[B7] Chamard E. Henry L. Boulanger Y. Lassonde M. Théoret H. (2014). A follow-up study of neurometabolic alterations in female concussed athletes. J. Neurotrauma 31, 339–345. 10.1089/neu.2013.3083 24053210

[B8] Chamard E. Lassonde M. Henry L. Tremblay J. Boulanger Y. De Beaumont L.. (2013). Neurometabolic and microstructural alterations following a sports-related concussion in female athletes. Brain Injury 27, 1038–1046. 10.3109/02699052.2013.794968 23834633

[B9] Chamard E. Théoret H. Skopelja E. N. Forwell L. A. Johnson A. M. Echlin P. S. (2012). A prospective study of physician-observed concussion during a varsity university hockey season: metabolic changes in ice hockey players. Neurosurg Focus 33, 1–7. 10.3171/2012.10.FOCUS12305 23199427

[B10] Churchill N. W. Hutchison M. G. Di Battista A. P. Graham S. J. Schweizer T. A. (2017). Structural, functional, and metabolic brain markers differentiate collision versus contact and non-contact athletes. Front. Neurol. 8, 1–11. 10.3389/fneur.2017.00390 28878729 PMC5572295

[B11] Dice L. R. (1945). Measures of the amount of ecologic association between species. Ecology. 26, 297–302. 10.2307/1932409

[B12] Ethofer T. Mader I. Seeger U. Helms G. Erb M. Grodd W.. (2003). Comparison of longitudinal metabolite relaxation times in different regions of the human brain at 1.5 and 3 Tesla. Magnet. Resonance Med. 50, 1296–1301. 10.1002/mrm.10640 14648578

[B13] Gangolli M. Benetatos J. Esparza T. J. Fountain E. M. Seneviratne S. Brody D. L. (2019). Repetitive concussive and subconcussive injury in a human tau mouse model results in chronic cognitive dysfunction and disruption of white matter tracts, but not tau pathology. J. Neurotrauma 36, 735–755. 10.1089/neu.2018.5700 30136628 PMC6387572

[B14] Ganji S. K. Banerjee A. Patel A. M. Zhao Y. D. Dimitrov I. E. Browning J. D.. (2012). T_2_ measurement of J-coupled metabolites in the human brain at 3T. NMR Biomed. 25, 523–529. 10.1002/nbm.1767 21845738 PMC3852663

[B15] Gasparovic C. Song T. Devier D. Bockholt H. J. Caprihan A. Mullins P. G.. (2006). Use of tissue water as a concentration reference for proton spectroscopic imaging. Magnet. Resonance Med. 55, 1219–1226. 10.1002/mrm.20901 16688703

[B16] Giza C. C. Hovda D. A. (2014). The new neurometabolic cascade of concussion. Neurosurgery 75, S24–S33. 10.1227/NEU.0000000000000505 25232881 PMC4479139

[B17] Goncalves S. Stevens T. K. Doyle-Pettypiece P. Bartha R. Duggal N. (2016). N -acetylaspartate in the motor and sensory cortices following functional recovery after surgery for cervical spondylotic myelopathy. J. Neurosurg. 25, 436–443. 10.3171/2016.2.SPINE15944 27176111

[B18] Gonzales M. M. Tarumi T. Kaur S. Nualnim N. Fallow B. A. Pyron M.. (2013). Aerobic fitness and the brain: increased N-acetyl-aspartate and choline concentrations in endurance-trained middle-aged adults. Brain Topogr. 26, 126–134. 10.1007/s10548-012-0248-8 22926147 PMC3537918

[B19] Guidelines for Data Processing and Analysis of the International Physical Activity Questionnaire (IPAQ) - Short and Long Forms (2005).

[B20] Haff G. G. Triplett N. T. (eds) (2015). Essentials of Strength Training and Conditioning, 4rth Edn. Champaign, IL: Human Kinetics.

[B21] Harris A. D. Puts N. A. J. Edden R. A. E. (2015). Tissue correction for GABA-edited MRS: considerations of voxel composition, tissue segmentation, and tissue relaxations. J. Magnet. Resonance Imaging 42, 1431–1440. 10.1002/jmri.24903 26172043 PMC4615266

[B22] Henry L. C. Tremblay S. Boulanger Y. Ellemberg D. Lassonde M. (2010). Neurometabolic changes in the acute phase. J. Neurotra. 27, 65–76. 10.1089/neu.2009.0962 19761385

[B23] Henry L. C. Tremblay S. Leclerc S. Khiat A. Boulanger Y. Ellemberg D.. (2011). Metabolic changes in concussed American football players during the acute and chronic post-injury phases. BMC Neurol. 11:105. 10.1186/1471-2377-11-105 21861906 PMC3176163

[B24] Huang R. Lu M. Song Z. Wang J. (2015). Long-term intensive training induced brain structural changes in world class gymnasts. Brain Struct. Funct. 220, 625–644. 10.1007/s00429-013-0677-5 24297657

[B25] King D. Hume P. Gissane C. Brughelli M. Clark T. (2016). The influence of head impact threshold for reporting data in contact and collision sports: systematic review and original data analysis. Sports Med. 46, 151–169. 10.1007/s40279-015-0423-7 26545363

[B26] King D. Hume P. A. Brughelli M. Gissane C. (2015). Instrumented mouthguard acceleration analyses for head impacts in amateur rugby union players over a season of matches. Am. J. Sports Med. 43, 614–624. 10.1177/0363546514560876 25535096

[B27] King D. A. Hume P. A. Gissane C. Kieser D. C. Clark T. N. (2018). Head impact exposure from match participation in women's rugby league over one season of domestic competition. J. Sci. Med. Sport 21, 139–146. 10.1016/j.jsams.2017.10.026 29122475

[B28] Koerte I. K. Ertl-Wagner B. Reiser M. Zafonte R. Shenton M. E. (2012). White matter integrity in the brains of professional soccer players without a symptomatic concussion. JAMA 308, 1859–1861. 10.1001/jama.2012.13735 23150002 PMC4103415

[B29] Koerte I. K. Lin A. P. Muehlmann M. Merugumala S. Liao H. Starr T.. (2015). Altered neurochemistry in former professional soccer players without a history of concussion. J. Neurotrauma 32, 1287–1293. 10.1089/neu.2014.3715 25843317 PMC4545372

[B30] Kreis R. (2016). The trouble with quality filtering based on relative Cramér-Rao lower bounds. Magnet. Resonance Med. 75, 15–18. 10.1002/mrm.25568 25753153

[B31] Lefebvre G. Chamard E. Proulx S. Tremblay S. Halko M. Soman S.. (2018). Increased myo-inositol in primary motor cortex of contact sports athletes without a history of concussion. J. Neurotrauma 35, 953–962. 10.1089/neu.2017.5254 29279021 PMC5865614

[B32] Lu H. Nagae-Poetscher L. M. Golay X. Lin D. Pomper M. Van Zijl P. C. M. (2005). Routine clinical brain MRI sequences for use at 3.0 tesla. J. Magnet. Resonance Imaging 22, 13–22. 10.1002/jmri.20356 15971174

[B33] Maddock R. J. Casazza G. A. Buonocore M. H. Tanase C. (2011). Vigorous exercise increases brain lactate and Glx (glutamate+glutamine): a dynamic 1H-MRS study. NeuroImage 57, 1324–1330. 10.1016/j.neuroimage.2011.05.048 21640838

[B34] Maddock R. J. Casazza G. A. Fernandez D. H. Maddock M. I. (2016). Acute modulation of cortical glutamate and GABA content by physical activity. J Neurosci. 36, 2449–2457. 10.1523/JNEUROSCI.3455-15.2016 26911692 PMC6705493

[B35] Manning K. Y. Brooks J. S. Dickey J. P. Harriss A. Fischer L. Jevremovic T.. (2020). Longitudinal changes of brain microstructure and function in nonconcussed female rugby players. Neurology 95:e402–e412. 10.1212/WNL.0000000000009821 32554762 PMC7455316

[B36] Manning K. Y. Llera A. Dekaban G. A. Bartha R. Barreira C. Brown A.. (2019). Linked MRI signatures of the brain's acute and persistent response to concussion in female varsity rugby players. NeuroImage 21, 1016–1027. 10.1016/j.nicl.2018.101627 30528959 PMC6411783

[B37] Martini D. N. Broglio S. P. (2018). Long-term effects of sport concussion on cognitive and motor performance: a review. Int J Psychophysiol. 132, 25–30. 10.1016/j.ijpsycho.2017.09.019 29017781

[B38] Mayer A. R. Ling J. M. Dodd A. B. Gasparovic C. Klimaj S. D. Meier T. B. (2015). A longitudinal assessment of structural and chemical alterations in mixed martial arts fighters. J. Neurotrauma 32, 1759–1767. 10.1089/neu.2014.3833 26096140

[B39] McCrory P. Meeuwisse W. H. Aubry M. Cantu R. C. Dvorák J. Echemendia R. J.. (2013). Consensus statement on concussion in sport: the 4th International Conference on Concussion in Sport, Zurich, November 2012. J. Athletic Train. 48, 554–575. 10.4085/1062-6050-48.4.05 23855364 PMC3715021

[B40] McGroarty N. K. Brown S. M. Mulcahey M. K. (2020). Sport-related concussion in female athletes a systematic review. Orthopaed. J. Sports Med. 8, 1–12. 10.1177/2325967120932306 32728590 PMC7366411

[B41] Mlynarik V. Gruber S. Moser E. (2001). Proton T1 and T2 relaxation times of human brain metabolites at 3 Tesla. NMR Biomed. 14, 325–331. 10.1002/nbm.713 11477653

[B42] Montenigro P. H. Alosco M. L. Martin B. M. Daneshvar D. H. Mez J. Chaisson C. E.. (2017). Cumulative head impact exposure predicts later-life depression, apathy, executive dysfunction, and cognitive impairment in former high school and college football players. J. Neurotrauma 34, 328–340. 10.1089/neu.2016.4413 27029716 PMC5220530

[B43] Panchal H. Sollmann N. Pasternak O. Alosco M. L. Kinzel P. Kaufmann D.. (2018). Neuro-metabolite changes in a single season of university ice hockey using magnetic resonance spectroscopy. Front. Neurol. 9:616. 10.3389/fneur.2018.00616 30177905 PMC6109794

[B44] Piechnik S. K. Evans J. Bary L. H. Wise R. G. Jezzard P. (2009). Functional changes in CSF volume estimated using measurement of water T2 relaxation. Magnet. Resonance Med. 61, 579–586. 10.1002/mrm.21897 19132756

[B45] Poole V. N. Abbas K. Shenk T. E. Breedlove E. L. Breedlove K. M. Robinson M. E.. (2014). MR spectroscopic evidence of brain injury in the non-diagnosed collision sport athlete. Dev. Neuropsychol. 39, 459–573. 10.1080/87565641.2014.940619 25144258

[B46] Poole V. N. Breedlove E. L. Shenk T. E. Abbas K. Robinson M. E. Leverenz L. J.. (2015). Sub-concussive hit characteristics predict deviant brain metabolism in football athletes. Dev. Neuropsychol. 40, 12–17. 10.1080/87565641.2014.984810 25649774

[B47] Schranz A. L. Manning K. Y. Dekaban G. A. Fischer L. Jevremovic T. Blackney K.. (2018). Reduced brain glutamine in female varsity rugby athletes after concussion and in non-concussed athletes after a season of play. Human Brain Mapp. 39:1489–1499. 10.1002/hbm.23919 29271016 PMC6866259

[B48] Stanisz G. J. Odrobina E. E. Pun J. Escaravage M. Graham S. J. Bronskill M. J.. (2005). T1, T2 relaxation and magnetization transfer in tissue at 3T. Magnet. Resonance Med. 54, 507–512. 10.1002/mrm.20605 16086319

[B49] Träber F. Block W. Lamerichs R. Gieseke J. Schild H. H. (2004). 1H metabolite relaxation times at 3.0 Tesla: measurements of T1 and T2 values in normal brain and determination of regional differences in transverse relaxation. J. Magnet. Resonance Imaging 19, 537–545. 10.1002/jmri.20053 15112302

[B50] Tremblay S. Beaulé V. Proulx S. Tremblay S. Marjańska M. Doyon J.. (2014). Multimodal assessment of primary motor cortex integrity following sport concussion in asymptomatic athletes. Clin. Neurophysiol. 125, 1371–1379. 10.1016/j.clinph.2013.11.040 24462505 PMC4381958

[B51] Tremblay S. De Beaumont L. Henry L. C. Boulanger Y. Evans A. C. Bourgouin P.. (2013). Sports concussions and aging: a neuroimaging investigation. Cereb. Cortex 23:1159–1166. 10.1093/cercor/bhs102 22581847

[B52] Wang B. Fan Y. Lu M. Li S. Song Z. Peng X.. (2013). Brain anatomical networks in world class gymnasts: a DTI tractography study. NeuroImage 65, 476–487. 10.1016/j.neuroimage.2012.10.007 23073234

[B53] Wansapura J. P. Holland S. K. Dunn R. S. Ball W. S. (1999). NMR relaxation times in the human brain at 3.0 Tesla. J. Magnet. Resonance Imag. 9, 531–538. 10.1002/(SICI)1522-2586(199904)9:4<531::AID-JMRI4>3.0.CO;2-L 10232510

[B54] Wong D. Schranz A. L. Bartha R. (2018). Optimized in vivo brain glutamate measurement using long-echo-time semi-LASER at 7 T. NMR Biomed. 31, 1–13. 10.1002/nbm.4002 30144183

[B55] Wyss P. O. Bianchini C. Scheidegger M. Giapitzakis I. A. Hock A. Fuchs A.. (2018). *In vivo* estimation of transverse relaxation time constant (T 2) of 17 human brain metabolites at 3T. Magnet. Resonance Med. 80, 452–461. 10.1002/mrm.27067 29344979

[B56] Zhang Y. Shen J. (2016). Simultaneous quantification of glutamate and glutamine by j-modulated spectroscopy at 3 Tesla. Magn. Reson. Med. 76, 725–732. 10.1002/mrm.25922 26361892 PMC4788985

