# Supplementary material for: Brain Metabolite Levels in Sedentary Women and Non-contact Athletes Differ From Contact Athletes
Source: Front Hum Neurosci. 2020 Nov 26;14:593498. doi: 10.3389/fnhum.2020.593498 (PMC7726472; doi:10.3389/fnhum.2020.593498)
Supplement: Supplementary file 1 [file Data_Sheet_1.docx]

Supplementary Material

# Supplementary Figures and Tables

## Supplementary Figures

**Supplementary Figure 1.** Initially a one-way ANOVA was used to test for any differences across all 4 groups. Then, a two-way ANOVA was used to test for differences between rowers and swimmers at both measurement times, before consolidating the teams into the non-contact group. Then the one-way ANOVA across all groups was repeated, to test for differences across contact, non-contact, and sedentary groups at the end of season. Finally, a two-way ANOVA was used to test for differences between contact and non-contact athletes. A *p*-value of 0.05 was used for all statistical tests.

**Supplementary Figure 2:** Mean In-Season Concentration of *N*-acetyl aspartate (Top) and Glutamate (Bottom). Rowers and Swimmers make up the Non-Contact group (Left). No significance was observed in *N*-acetyl aspartate between groups in either analysis. Glutamate is significantly different from Contact Rugby athletes in both analyses.
